# Genomic characterization of triple-carbapenemase-producing *Acinetobacter baumannii*

**DOI:** 10.1093/jacamr/dlab191

**Published:** 2021-12-18

**Authors:** Ken-Ichi Oinuma, Masato Suzuki, Arata Sakiyama, Taishi Tsubouchi, Kozo Saeki, Kanako Sato, Mamiko Niki, Koichi Yamada, Keigo Shibayama, Hiroshi Kakeya, Yukihiro Kaneko

**Affiliations:** 1 Department of Bacteriology, Osaka City University Graduate School of Medicine, 1-4-3 Asahimachi, Abeno-ku, Osaka 545-8585, Japan; 2 Research Center for Infectious Disease Sciences, Osaka City University Graduate School of Medicine, 1-4-3 Asahimachi, Abeno-ku, Osaka 545-8585, Japan; 3 Antimicrobial Resistance Research Center, National Institute of Infectious Diseases, 4-2-1 Aoba-cho, Higashimurayama, Tokyo 189-0002, Japan; 4 Department of Medical Technology, Morinomiya University of Medical Sciences, 1-26-16 Nankokita, Suminoe-ku, Osaka 559-8611, Japan; 5 Department of Infection Control Science, Osaka City University Graduate School of Medicine, 1-4-3 Asahimachi, Abeno-ku, Osaka 545-8585, Japan; 6 Department of Bacteriology, Nagoya University Graduate School of Medicine, 65 Tsurumai-cho, Showa-ku, Nagoya 466-8550, Japan

## Abstract

**Objectives:**

To characterize *Acinetobacter baumannii* OCU_Ac16a, a clinical isolate co-harbouring three acquired carbapenemase genes, *bla*_NDM-1_, *bla*_TMB-1_, and *bla*_OXA-58_, and assess the clinical significance of so-called multiple-carbapenemase producers.

**Methods:**

OCU_Ac16a and its close relative, OCU_Ac16b, which lacks the *bla*_NDM-1_, were isolated from sputum cultures of a patient at Osaka City University Hospital. We subjected these strains to whole-genome analysis, particularly focusing on the genetic context of each carbapenemase gene. The transmissibility and functionality of each carbapenemase gene were analysed by conjugation and transformation experiments and antimicrobial susceptibility tests.

**Results:**

*bla*_TMB-1_ was located in a class 1 integron on the chromosome, whereas *bla*_NDM-1_ and *bla*_OXA-58_ were found on plasmids named pOCU_Ac16a_2 and pOCU_Ac16a_3, respectively. pOCU_Ac16a_2 (which exhibited highly efficient self-transmissibility) and pOCU_Ac16a_3 (which did not show transmissibility but could be introduced into another *A. baumannii* strain via electroporation) could both confer carbapenem resistance (MICs ≥512 and ≥32 mg/L, respectively) on the recipient strain. The functionality of *bla*_TMB-1_ was evident from the high resistance of OCU_Ac16b to ceftazidime and cefepime (MICs ≥256 and 48 mg/L, respectively), and the high resistance of OCU_Ac16a to cefiderocol (MIC 32 mg/L) could be explained by the additive effect of *bla*_NDM-1_ and *bla*_TMB-1_.

**Conclusions:**

Our data revealed the genomic organization of OCU_Ac16a and demonstrated that all the carbapenemase genes are functional, each contributing to the extremely high broad-spectrum resistance of OCU_Ac16a to β-lactams. As multiple-carbapenemase producers can be serious health threats as drug-resistant pathogens and disseminators of carbapenemase genes, close attention should be paid to their emergence.

## Introduction

The increase in antimicrobial-resistant (AMR) bacteria is posing a serious threat to human health worldwide. One such AMR bacterial species is *Acinetobacter baumannii*, which has acquired clinically relevant AMR genes, such as carbapenemase genes, owing to the horizontal gene transfer of mobile genetic elements, such as plasmids.^1^ The carbapenem antimicrobials are used as a last resort against Gram-negative bacterial infections. However, they have become less effective due to the global spread of carbapenemase genes.^2^^,^^3^ In *Acinetobacter* spp., the most common group of carbapenemases is Ambler’s class D, which consists of enzymes referred to as oxacillinases (OXAs). In addition, Ambler’s class B, consisting of metallo-β-lactamases [e.g. New Delhi metallo-β-lactamase (NDM) and Tripoli metallo-β-lactamase (TMB)],^4^^,^^5^ is also prevalent.

Notably, over the past decade, a significant number of studies have reported the emergence of bacterial strains that simultaneously possess two different carbapenemase genes. While the emergence of triple-carbapenemase producers has also been reported,^6^^,^^7^ no studies have attempted to perform a thorough characterization including complete genome sequencing and assessment of the functionality of each carbapenemase gene. Multiple-carbapenemase producers pose a more serious health risk than others given that they are more difficult to combat and can act as a reservoir of carbapenemase genes for other pathogens. Therefore, evaluating the consequences of their prevalence and addressing the mechanism of how and why they emerge in clinical settings is necessary.

We recently identified a carbapenem-resistant *A. baumannii* strain, OCU_Ac16a that was isolated from the intratracheal aspirate of a patient with oesophageal cancer at Osaka City University Hospital in Japan in 2015.^8^ The analysis of this strain via draft genome sequencing and subsequent multilocus sequence typing (MLST) revealed that this strain belonged to sequence type 412 (ST412) and co-harboured three acquired carbapenemase genes, namely, *bla*_NDM-1_, *bla*_TMB-1_, and *bla*_OXA-58_, in addition to the intrinsic *bla*_OXA-51_-like and *bla*_ADC-25_-like β-lactamase genes. It should be noted that two of these genes encode metallo-β-lactamases (NDM-1 and TMB-1). Post isolation of OCU_Ac16a, a possible variant of OCU_Ac16a (named OCU_Ac16b), co-harbouring *bla*_TMB-1_ and *bla*_OXA-58_, but not *bla*_NDM-1_, was isolated from the same patient. In this study, we aimed to elucidate their genomic organization and also assess the impact of each carbapenemase gene on carbapenem resistance in order to evaluate the clinical significance of the emerging strains called multiple-carbapenemase producers.

## Materials and methods

### Ethics

The study conformed to the principles of the Declaration of Helsinki and was approved by the Institutional Ethics Review Board (approval no. 3568, 9/30/2016). Informed consent was waived according to the ethical guidelines for human research in Japan.

### Clinical setting and isolation of bacterial strains

The *A. baumannii* strains OCU_Ac16a and OCU_Ac16b were isolated from a patient with type 3 oesophageal cancer in the middle thoracic oesophagus. The patient underwent transthoracic oesophagectomy followed by gastric tube reconstruction. OCU_Ac16a was isolated from suctioned sputum culture on postoperative day (POD) 32, whereas OCU_Ac16b was isolated from sputum obtained by bronchoscopy on POD 35 (for more detail on the methods see Supplementary data, available at *JAC* Online).

### Antimicrobial susceptibility tests

Antimicrobial susceptibility tests were conducted in accordance with the criteria specified by the Clinical and Laboratory Standards Institute^9^ using the broth microdilution method or Etest (bioMérieux Inc., Marcy-l’Etoile, France). Cation-adjusted Mueller Hinton (CAMH) broth or agar plates were used for all the tests except broth microdilution tests with cefiderocol, which were performed using iron-depleted CAMH broth. OCU_Ac16b susceptibility to cefiderocol could not be determined because of a growth defect that occurred in the iron-depleted CAMH broth. Therefore, we additionally performed a disc diffusion assay with MASTDISCS AST Cefiderocol 30 μg (Mast Group Ltd., Merseyside, UK) using non-iron-depleted CAMH agar plates.

### Genome sequencing and analyses

Whole-genome sequencing of OCU_Ac16a and OCU_Ac16b were performed using the MiSeq system (Illumina, San Diego, CA) as previously described.^8^ OCU_Ac16a was further sequenced using the PacBio RS II system (Pacific Biosciences, Menlo Park, CA) so as to construct the complete genome sequence, including the plasmid sequences (for method details, see Supplementary data). The sequences of complete chromosomal DNA, pOCU_Ac16a_1, pOCU_Ac16a_2, and pOCU_Ac16a_3 have been deposited in the DDBJ/EMBL/GenBank databases under accession numbers AP023077–AP023080. Whole-genome shotgun assembly of the OCU_Ac16b genome has been deposited under accession numbers BLWH01000001–BLWH01000743.

MLST was performed using the Institut Pasteur MLST scheme (http://pubmlst.org/abaumannii/). Antimicrobial resistance genes were detected using ResFinder v3.2 (http://cge.cbs.dtu.dk/services/ResFinder/). Genetic elements related to plasmid mobility were detected using the web-based tool, oriTfinder.^10^ The classification of relaxases and mating pair formation systems was realized based on a scheme that was previously described by Smillie *et al*.^11^ Plasmid replicon typing was performed based on the *rep* gene sequence.^12^

### Conjugation experiments

Conjugation experiments were performed using OCU_Ac16a as the donor and spontaneous rifampicin-resistant mutants (RFP50R) of *A. baumannii* ATCC 19606^T^, *Acinetobacter ursingii* OCU_Ac4, *Acinetobacter soli* OCU_Ac8 and OCU_Ac9, and *Acinetobacter pittii* OCU_Ac12^13^ as recipients (refer to Supplementary data for details). Transconjugants were selected on CAMH plates containing rifampicin and/or meropenem (50 mg/L each). The conjugal transfer frequency was defined as the ratio of the number of transconjugant cells that grew on plates containing both rifampicin and meropenem to the total number of recipient cells that grew on rifampicin-containing plates.

## Results and discussion

The complete genome of OCU_Ac16a consisted of a chromosome and three plasmids, named pOCU_Ac16a_1, pOCU_Ac16a_2, and pOCU_Ac16a_3, with characteristics as summarized in Table 1. It was shown that pOCU_Ac16a_1 and pOCU_Ac16a_3 belonged to homology groups 6 and 4, respectively. However, pOCU_Ac16a_2 was untypeable owing to the lack of an apparent *rep*-like gene. *bla*_NDM-1_ and *bla*_OXA-58_ were found to be located on pOCU_Ac16a_2 and pOCU_Ac16a_3, respectively, whereas *bla*_TMB-1_ was found to be located on the chromosome (Table 1). All the remaining AMR genes were found to be located on the chromosome, except for *aph(3′)-VIa*, which was located on pOCU_Ac16a_2. The OCU_Ac16b draft genome consisted of 743 contigs, and MLST analysis revealed that this isolate belonged to the same sequence type (ST412) as OCU_Ac16a, indicating their clonality. Additionally, a comparative analysis involving the genome sequences showed that OCU_Ac16b was nearly identical to OCU_Ac16a, except that it lacked the whole pOCU_Ac16a_2 plasmid.

**Table 1.** Characteristics of replicons identified in OCU_Ac16a

Replicon	Size (bp)	Antimicrobial resistance genes	*rep* gene group of plasmids	Elements related to plasmid mobility	Frequencies of plasmid transfer^a^
Chromosome	3 992 063	*bla*_TMB-1_, *bla*_ADC-25_-like, *bla*_OXA-51_-like, *strA*, *strB*, *mph(E)*, *msr(E)*, *sul1*, *sul2*, *gyrA* (S81L)	–	–	–
pOCU_Ac16a_1	73 028	ND	GR6	MOB_F_ family relaxase, TraD-like T4CP, MPF_F_	–
pOCU_Ac16a_2	41 087	*bla*_NDM-1_, *aph(3′)-VIa*	ND	*oriT*, MOB_Q_ family relaxase, TrwB-like T4CP, MPF_T_	2.5 × 10^−5^–1.5 × 10^−2^ (*A. baumannii* ATCC 19606^T^ RFP50R)^b^; 2.2 × 10^−7^–5.4 × 10^−1^ (*Acinetobacter* strains OCU_Ac4, 8, 9, and 12 RFP50R)
pOCU_Ac16a_3	13 096	*bla*_OXA-58_	GR4	ND	ND (*A. baumannii* ATCC 19606^T^ RFP50R)

ND, not detected; –, not applicable or not tested; GR, homology group; MOB, mobilization; T4CP, type IV coupling protein; MPF, mating pair formation; *oriT*, origin of transfer.

a The recipient strains used are indicated in parentheses.

b Results from four independent experiments.

OCU_Ac16a was highly resistant to all the β-lactam antimicrobials tested, including cefiderocol, a novel siderophore cephalosporin (MICs ≥256 mg/L for piperacillin, ceftazidime, and cefepime, ≥512 mg/L for imipenem and meropenem, and 32 mg/L for cefiderocol; 6 mm zone of inhibition with cefiderocol discs). However, it was susceptible to gentamicin, amikacin, levofloxacin, colistin, minocycline, and tigecycline (MICs 1, 8, 1, 0.094, 0.031, and 0.25 mg/L, respectively) (Table 2). The antimicrobial susceptibility pattern of OCU_Ac16b was very similar to that of OCU_Ac16a, although OCU_Ac16b was more susceptible to cefepime, cefiderocol, and amikacin (MICs 48 and 3 mg/L for cefepime and amikacin, respectively; 21 mm zone of inhibition with cefiderocol discs) (Table 2).

**Table 2.** Antimicrobial susceptibility of strains used in this study^a^

Strain	Antimicrobials											
	PIP	CAZ	FEP	IPM	MEM	FDC	GEN	AMK	LVX	CST	MIN	TGC
OCU_Ac16a	≥256 (R)	≥256 (R)	≥256 (R)	≥512 (R)	≥512 (R)	32 (R); 6 mm (R)	1 (S)	8 (S)	1 (S)	0.094 (S)	0.031 (S)	0.25 (S)
OCU_Ac16b	≥256 (R)	≥256 (R)	48 (R)	≥512 (R)	≥512 (R)	NA; 21 mm (S)	0.75 (S)	3 (S)	1.5 (S)	0.094 (S)	0.031 (S)	0.25 (S)
ATCC 19606^T^ RFP50R	24 (I)	4 (S)	8 (S)	0.5 (S)	2 (S)	≤0.031 (S); 28 mm (S)	12 (I)	16 (S)	0.38 (S)	0.094 (S)	NT	NT
ATCC 19606^T^ RFP50R with pOCU_Ac16a_2	≥256 (R)	≥256 (R)	≥256 (R)	≥512 (R)	≥512 (R)	0.5 (S); 20 mm (S)	8 (I)	192 (R)	0.25 (S)	0.094 (S)	NT	NT
ATCC 19606^T^ RFP50R with pOCU_Ac16a_3	≥256 (R)	4 (S)	8 (S)	64 (R)	32 (R)	≤0.031 (S); 29 mm (S)	NT	NT	NT	NT	NT	NT

PIP, piperacillin; CAZ, ceftazidime; FEP, cefepime; IPM, imipenem; MEM, meropenem; FDC, cefiderocol; GEN, gentamicin; AMK, amikacin; LVX, levofloxacin; CST, colistin; MIN, minocycline; TGC, tigecycline; S, susceptible; I, intermediate; R, resistant; NA, not applicable; NT, not tested.

a MICs (mg/L) determined by performing Etest (for PIP, CAZ, FEP, GEN, AMK, LVX, and CST) or employing the microdilution method (for IPM, MEM, FDC, MIN, and TGC). The observed diameters of the zone of inhibition from FDC disc diffusion assays are shown.

Subsequently, we investigated the genetic contexts of the three acquired carbapenemase genes (Figure S1). *bla*_TMB-1_ was found to be located in a class 1 integron and was similar to its counterpart in *A. baumannii* strain A1, which was the first *Acinetobacter* strain clinically isolated in Japan in 2009 reported to possess *bla*_TMB-1_.^14^*bla*_NDM-1_ was found to be located in a cluster with high overall identity with species previously reported to carry this gene (Figure S2).^15–17^ The complete conservation of the promoter sequence, which is partly constituted from the right end of IS*Aba125* located upstream of the *bla*_NDM-1_ structural gene, was confirmed. The cluster containing *bla*_OXA-58_ was similar to the plasmid, pTVICU14, from the *Acinetobacter nosocomialis* strain TVICU14 that was clinically isolated in Taiwan in 2004,^18^ whereas pOCU_Ac16a_3 showed no overall identity with any plasmid in the NCBI database. A promoter-like sequence that is composed of the right end of IS*1008* and a part of ΔIS*Aba3*-like sequence, highly similar to that in pTVICU14, was observed in the upstream region of *bla*_OXA-58_ in OCU_Ac16a (Figure S1).

pOCU_Ac16a_1 and pOCU_Ac16a_2 were possibly self-transmissible given that they contained a set of mobilization elements. Our conjugation experiments further demonstrated the transmissibility of pOCU_Ac16a_2 (Table 1). pOCU_Ac16a_2 caused a significant increase in resistance to β-lactams, including carbapenems, and amikacin in *A. baumannii* ATCC 19606^T^ RFP50R (MICs ≥256 mg/L for piperacillin, ceftazidime, and cefepime, ≥512 mg/L for imipenem and meropenem, and 192 mg/L for amikacin) (Table 2). Notably, however, this transconjugant was susceptible to cefiderocol (MIC 0.5 mg/L; 20 mm zone of inhibition with cefiderocol discs), an observation that is consistent with previous studies that reported the efficacy of cefiderocol against NDM-1-producing bacteria.^19^

Although we did not observe the transfer of pOCU_Ac16a_3 or *bla*_OXA-58_ in our conjugation experiments, we successfully transformed the plasmid into *A. baumannii* ATCC 19606^T^ RFP50R via electroporation. The transformant showed significant resistance to piperacillin, imipenem, and meropenem (MICs ≥256, 64, and 32 mg/L, respectively) (Table 2). These data clearly demonstrate that *bla*_NDM-1_ and *bla*_OXA-58_ in pOCU_Ac16a_2 and pOCU_Ac16a_3 are functional. Additionally, we reasoned that *bla*_TMB-1_ is functional because OCU_Ac16b shows significant resistance to ceftazidime and cefepime, which are β-lactams to which *bla*_OXA-58_ does not usually confer resistance.^20^ Of note, no insertion sequence was detected upstream of the *bla*_ADC-25_-like gene, which rules out the theory that overexpression of this intrinsic cephalosporinase caused the high resistance to ceftazidime and cefepime in OCU_Ac16b.

We found it particularly interesting that OCU_Ac16a was resistant to cefiderocol despite the fact that oxacillinases and metallo-β-lactamases do not generally confer resistance to this drug.^19^ We assume this was caused by an additive effect of NDM-1 and TMB-1, both of which have a certain level of ability to increase resistance of *A. baumannii* to cefiderocol. This is a good example that explains how challenging it is to combat multiple-carbapenemase producers. Although several promising β-lactamase inhibitors with efficacy against carbapenemases have been or are being developed, including avibactam and vaborbactam, they are less promising as a weapon against bacteria such as OCU_Ac16a because there are only a few candidate compounds that can inhibit metallo-β-lactamases and no one compound can universally inhibit multiple classes of β-lactamases.^21^ To prevent future public health crises, the appropriate use of antimicrobials is important and further studies on the mechanism behind multiple occurrences of AMR genes are necessary.

## Supplementary Material

dlab191_Supplementary_Data
