# Supplementary material for: Genomic characterization of triple-carbapenemase-producing Acinetobacter baumannii
Source: JAC Antimicrob Resist. 2021 Dec 18;3(4):dlab191. doi: 10.1093/jacamr/dlab191 (PMC8684466; doi:10.1093/jacamr/dlab191)
Supplement: dlab191_Supplementary_Data [file dlab191_supplementary_data.docx]

**Supplementary data**

**Methods**

**Clinical setting and isolation of bacterial strains**

This study was conducted at Osaka City University, Japan. The study conformed to the principles of the Declaration of Helsinki and was approved by the Institutional Ethics Review Board (approval no. 3568, 9/30/2016). Informed consent was waived according to the ethical guidelines for human research in Japan. The *A. baumannii* strains OCU_Ac16a and OCU_Ac16b were isolated from a patient with type 3 esophageal cancer in the middle thoracic esophagus. The patient underwent transthoracic esophagectomy followed by gastric tube reconstruction. OCU_Ac16a was isolated from suctioned sputum culture on postoperative day (POD) 32, whereas OCU_Ac16b was isolated from sputum obtained by bronchoscopy on POD 35. The patient received empirical antimicrobial therapy with cefozopran and tazobactam/piperacillin starting from POD 4 through POD 7 and from POD 7 through POD 16, respectively, to treat suspected pneumonia. Since a chest radiograph revealed a persistent bilateral pleural effusion with a new right lower lobe infiltrate, doripenem was administered from POD 27. However, this treatment was not effective, and thus the antimicrobial was switched to levofloxacin on POD 35. Although this treatment improved and stabilized the patient’s condition as well as eliminated the carbapenem-resistant *Acinetobacter* strains, the patient’s condition began worsening on POD 64 and he deceased on POD 86 because of sepsis followed by multiple organ failure.

**Genome sequencing and assembly**

Genomic DNA of *A. baumannii* was prepared using MagAttract HMW DNA Kit (Qiagen, Hilden, Germany). Whole-genome sequencing of *A. baumannii* isolates, OCU_Ac16a and OCU_Ac16b, was performed using the MiSeq system (Illumina, San Diego, CA) with MiSeq Reagent Kit v3. The draft genome sequences were obtained as described in a previous study.^1^ Further, we sequenced *A. baumannii* OCU_Ac16a using the PacBio RS II system (Pacific Biosciences, Menlo Park, CA) with the DNA sequencing reagent kit 4.0 v2 and SMRT Cell v3 to construct the complete genome consisting of the chromosome and plasmids. The library for PacBio sequencing (insert size of approximately 20 kbp) was prepared using the SMRTbell template prep kit v1.0. PacBio reads were basecalled and assembled *de novo* using SMRT Analysis Software v2.3.0 with the default parameters. Overlap region in the assembled contig was determined by a genome-scale sequence comparison using LAST (http://last.cbrc.jp) and was trimmed manually. Illumina reads were mapped onto the resulting circular chromosome and plasmids, and sequencing errors were corrected by extracting the consensus of the mapped reads using the CLC Genomics Workbench v11.0.1 (Qiagen). Coding genes were annotated using the RAST toolkit on the PATRIC server (https://www.patricbrc.org/).

**Conjugation and transformation experiments**

We used spontaneous rifampicin-resistant mutants (RFP50Rs) of *A. baumannii* ATCC 19606^T^, *A. ursingii* OCU_Ac4, *A. soli* OCU_Ac8 and OCU_Ac9, and *A. pittii* OCU_Ac12^2^ as recipients to assess the conjugal transfer frequency of the pOCU_Ac16a_2 plasmid. These mutants were obtained by first cultivating the parental rifampicin-susceptible strains in 1–10 mg/L of rifampicin-containing cation-adjusted Mueller Hinton (CAMH) liquid medium at 37°C overnight followed by subculturing in 50 mg/L of rifampicin-containing medium. To perform conjugation, 1% volume of an overnight culture of OCU_Ac16a (donor) was inoculated into 7 mL fresh LB medium, and cultivated at 30°C with shaking at 130 rpm until the optical density at 600 nm reached 0.1–0.2. Meanwhile, overnight cultures of the recipient strains (1 mL each) were mixed with 1 mL of fresh LB containing NaNO_3_ at a final concentration of 10 mM and incubated at 42°C for 4–5 h without shaking. The resulting donor and recipient cultures were mixed and centrifuged briefly in 1.5 mL tubes to collect the cells, which were then spread on CAMH agar plates and cultivated overnight at 30°C. Cells that grew on these plates were suspended in a small volume of phosphate-buffered saline, appropriately diluted, and spread on CAMH plates containing rifampicin and/or meropenem (50 mg/L each). After overnight cultivation at 35°C, colony counting was performed for each plate. The conjugal transfer frequency was defined as the ratio of the number of transconjugant cells that grew on plates containing both rifampicin and meropenem to the total number of recipient cells that grew on rifampicin-containing plates.

pOCU_Ac16a_3 was introduced into *A. baumannii* ATCC 19606^T^ RFP50R by electrotransformation with a plasmid sample prepared from OCU_Ac16a cells using QIAprep Spin Miniprep Kit (Qiagen). CAMH agar plates containing 200 mg/L of piperacillin were used for selection. Successful transformation was confirmed by PCR targeting *bla*_OXA-58_.

**References**

1. Oinuma K, Suzuki M, Sato K *et al*. Genome sequence of an *Acinetobacter baumannii* strain carrying three acquired carbapenemase genes. *Genome Announc* 2016; **4**: e01290–16.
2. Oinuma K, Suzuki M, Nakaie K *et al*. Genome-based epidemiological analysis of 13 *Acinetobacter* strains isolated from blood cultures of hospitalized patients from a university hospital in Japan. *Jpn J Infect Dis* 2019; **72**: 274–80.
3. Chen TL, Chang WC, Kuo SC *et al*. Contribution of a plasmid-borne *bla*_OXA-58_ gene with its hybrid promoter provided by IS*1006* and an IS*Aba3*-like element to β-lactam resistance in *Acinetobacter* genomic species 13TU. *Antimicrob Agents Chemother* 2010; **54**: 3107–12.
4. Dortet L, Nordmann P, Poirel L. Association of the emerging carbapenemase NDM-1 with a bleomycin resistance protein in *Enterobacteriaceae* and *Acinetobacter baumannii*. *Antimicrob Agents Chemother* 2012; **56**: 1693–7.


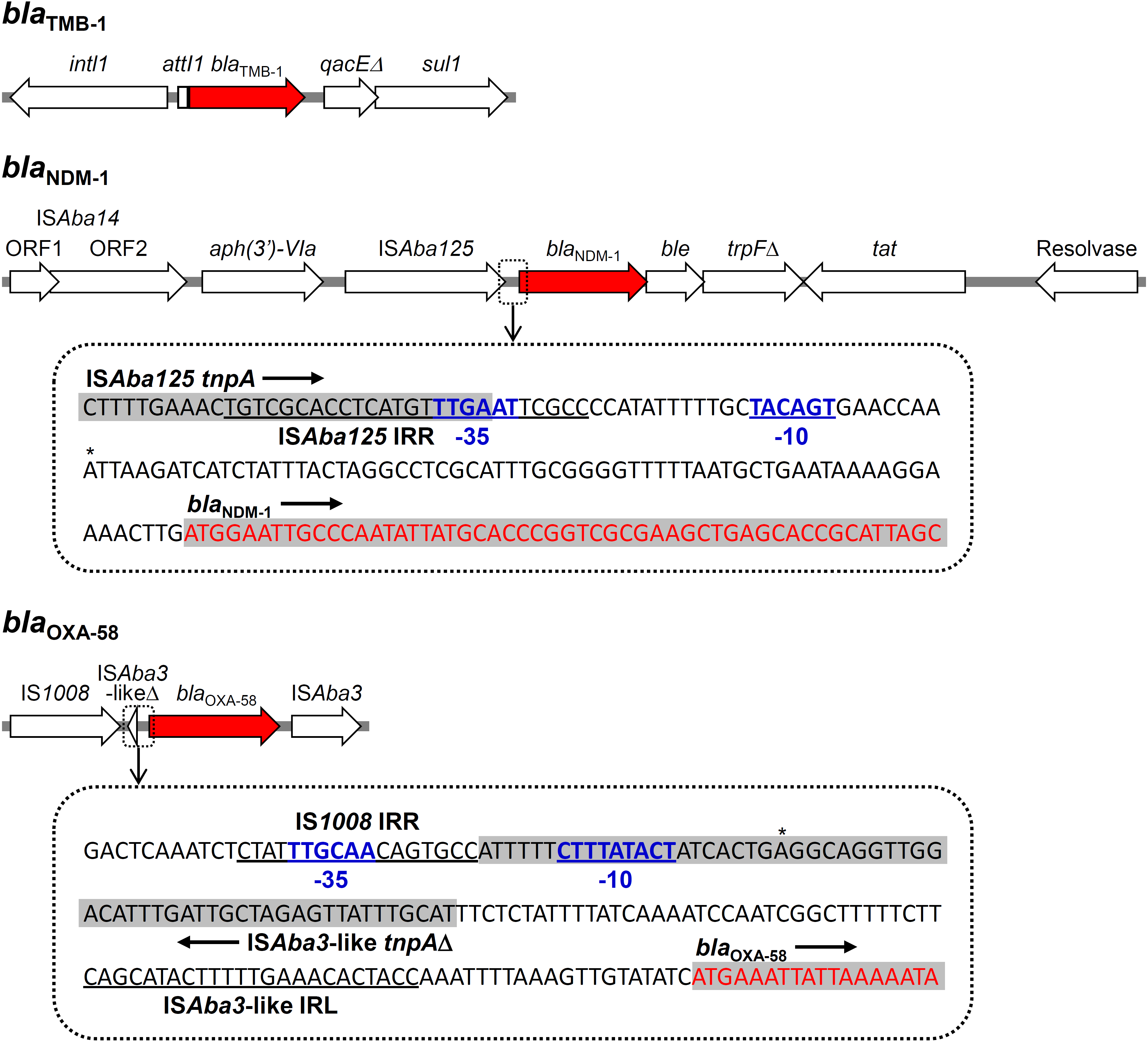


**Figure S1.** Genetic contexts of carbapenemase genes identified in the OCU_Ac16a genome. A schematic representation of gene clusters containing the indicated carbapenemase genes is shown. Arrows indicate the size, location, and transcriptional direction of the genes in the clusters. Dashed boxes below the schematics show the nucleotide sequences of the upstream regions of *bla*_NDM-1_ and *bla*_OXA-58_. Shaded letters indicate partial nucleotide sequences of *tnpA*, *bla*_NDM-1_, or *bla*_OXA-58_ structural genes. Carbapenemase genes and putative promoter regions (-35 and -10 elements) are shown in red and blue, respectively. Star indicates the +1 transcription start sites inferred based on published data.^3,4^ The right inverted repeat (IRR) and left inverted repeat (IRL) of insertion sequences are underlined.


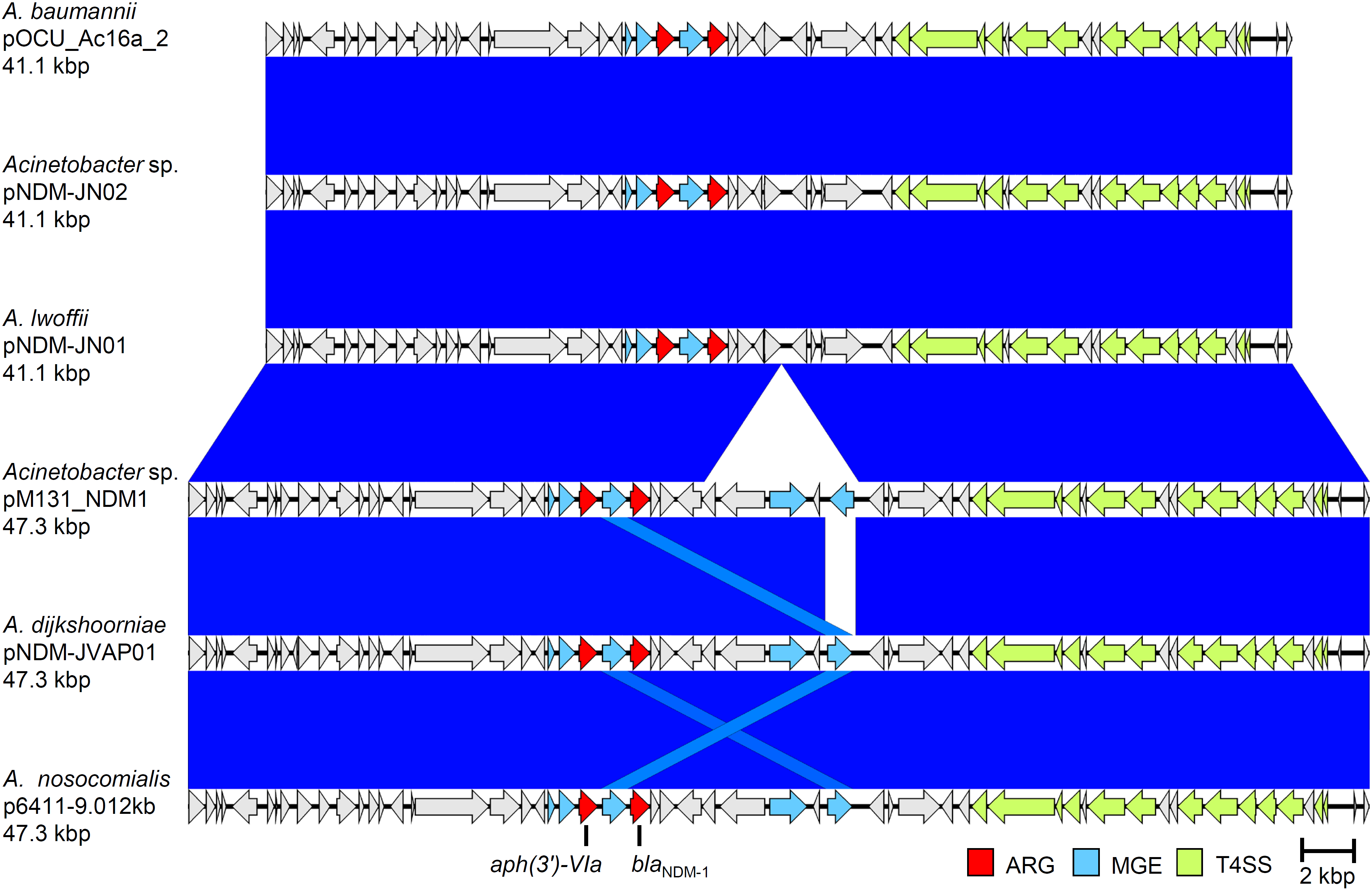


**Figure S2.** Comparison of pOCU_Ac16a_2 with other identical plasmids. The following plasmids were used in this analysis: pNDM-JN01 (GenBank ID: KM210086.1), pNDM-JN02 (GenBank ID: KM210088.1), pM131_NDM1 (GenBank ID: JX072963.1), pNDM-JVAP01 (GenBank ID: KM923969.1), and p6411-9.012kb (GenBank ID: CP010370.2), each isolated from different *Acinetobacter* spp. as indicated in the figure. Arrows indicate the ORFs identified by sequence analysis. Blue and light blue boxes drawn between gene cluster schematics indicate high sequence homology. Antimicrobial resistance genes (ARGs), mobile genetic elements (MGEs), and genes encoding the components of a type IV secretion system (T4SS) are indicated in red, light blue, and green, respectively. The bar indicates a scale of 2 kilobase pairs.
